# The association between variation of neutrophil-to-lymphocyte ratio and post-thrombolysis early neurological outcomes in patients with stroke of different TOAST classification

**DOI:** 10.1038/s41598-025-91334-z

**Published:** 2025-02-22

**Authors:** Xinyi Fu, Xinyan Shi, Ruihua Yin, Chengfeng Xing, Aijun Ma

**Affiliations:** Department of Neurology, The Affiliated Hospital of Qingdao University, Qingdao, 266000 China

**Keywords:** Neutrophil-to-lymphocyte ratio, Acute ischemic stroke, Intravenous thrombolysis, Hemorrhagic transformation, TOAST classification, Neuroscience, Diseases, Neurology

## Abstract

Recent studies have shown that the neutrophil-to-lymphocyte ratio (NLR) can predict short-term and long-term outcomes in acute ischemic stroke (AIS) patients. However, the relationship of the variation of NLR (ΔNLR) with hemorrhage transformation (HT) and early neurological improvement (ENI) after intravenous thrombolysis (IVT) remains unclear. This study aimed to investigate the impact of ΔNLR on HT and ENI at 24 h post-IVT and its association with different TOAST classifications. AIS patients undergoing IVT between October 2021 and October 2023 were enrolled and classified by TOAST criteria. Patients were grouped based on the presence or absence of HT and ENI. Our study demonstrated that both HT and ENI were associated with ΔNLR, which was an independent influencing factor for HT and ENI following IVT. Specifically, the ΔNLR in the small artery occlusion (SAO) group was higher than that in the minor stroke of large artery atherosclerosis (LAA) subtype. Thus, ΔNLR may serve as a useful biomarker to assist in diagnosis and monitor the outcomes of thrombolytic therapy in AIS patients.

## Introduction

Stroke is a condition that results in persistent disability and contributes to high mortality globally^1^. Multiple randomized controlled trials have demonstrated that administration of recombinant tissue plasminogen activator (rt-PA) within 4.5 h of stroke onset significantly improves the likelihood of a favorable prognosis, regardless of age or stroke severity^2^. However, hemorrhagic transformation (HT) is a serious complication of acute ischemic stroke (AIS), typically occurring within 24 h after thrombolysis. The complication can exacerbate the disease and significantly increase the disability and mortality rates at 3 months^3,4^. Therefore, predicting and analyzing HT is of great importance.

Neutrophils, early responders in the blood following AIS, contribute to ischemic brain injury, thrombosis, and atherosclerosis, while lymphocytes may protect against ischemic injury by suppressing inflammation^5,6^. The neutrophil-to-lymphocyte ratio (NLR) not only reflects the counts of neutrophils and lymphocytes but also indicates the over-activation of neutrophils, resulting in a larger disparity between these two subtypes of white blood cells^7^. The ratio offers advantages over studying only the two subtypes of leukocytes individually^7^. Previous studies have identified baseline NLR as an independent predictor of early neurological improvement (ENI), HT, and mortality in AIS patients^8–10^. Given that the counts of neutrophils and lymphocytes change dynamically in stroke^11,12^, the variation in NLR (ΔNLR) can reflect the progression of AIS patients after thrombolysis. However, the relationship between ΔNLR and post-thrombolysis early neurological outcomes has not been extensively studied.

We examined the relationship between ΔNLR and HT, as well as ENI following IVT in AIS patients. Additionally, given the scarcity of studies on the relationship between ΔNLR and TOAST classification, our study was the first to investigate the association between ΔNLR and various TOAST classifications.

## Participants and methods

### Study population

**Fig. 1:**
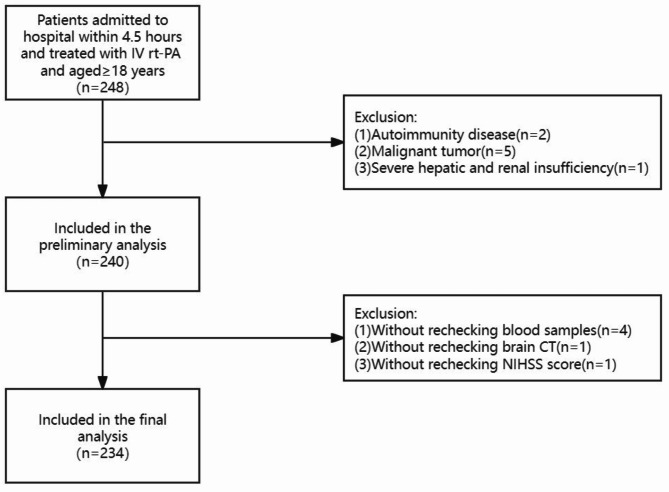
Flowchart of the selection of AIS patients

AIS patients undergoing intravenous thrombolysis within 4.5 h were recruited from the Department of Neurology, Affiliated Hospital of Qingdao University. All the patients were treated in the stroke units and received standard treatments, for instance, antiplatelet therapy and statin therapy. Informed consent was obtained from participants or their legal representatives. The study inclusion criteria were: (1) Admission within 4.5 h after onset; (2) Treated with intravenous thrombolysis; (3) Eighteen years or older. The study exclusion criteria were: (1) Autoimmunity disease, malignant tumor or severe hepatic and renal insufficiency; (2) Incomplete clinical data (Fig. 1). Stroke subtype was classified according to Trial of Org 10,172 in Acute Stroke Treatment (TOAST) criteria^13^.

### Data collection

Basic clinical data of all subjects, including gender, age, baseline National Institutes of Health Stroke Scale (NIHSS) score, onset to treatment time (OTT), admission systolic blood pressure, admission diastolic blood pressure, Body Mass Index (BMI) and vascular risk factors [hypertension, diabetes mellitus, dyslipidemia, coronary heart disease (CHD), atrial fibrillation, current smoking and current drinking] were obtained by two professional neurologists and two trained nurses. All patients underwent electrocardiogram, brain CT, brain MRI which included the sequences of T1, T2, Flair (fluid-attenuated inversion recovery), DWI (diffusion-weighted imaging), ADC (apparent diffusion coefficient)] and MRA (magnetic resonance angiography), chest CT, whole brain and cervical CT angiography (CTA) and digital subtraction angiography if it was necessary to determine the etiological classification. Patients fasted for at least 8 h before fasting blood collection. Routine biochemistry which included aspartate aminotransferase (AST), alanine aminotransferase (ALT), total bilirubin (TBIL), blood glucose (Glu), urea, creatinine, and C-reactive protein (CRP) and complete blood count analysis were obtained from the biochemistry Laboratory of Affiliated Hospital of Qingdao University. We calculated ΔNLR as follows: baseline NLR minus NLR after IVT. The complete blood count analysis, brain CT and the NIHSS score was performed again at 24 h following IVT. Modified Rankin Scale (mRS) score was performed at 7 days following IVT. We calculated the variation of NIHSS score (ΔNIHSS score) as follows: baseline NIHSS score minus NIHSS score after IVT. Post-thrombolysis ENI was defined as a decrease in the NIHSS score by ≥ 4 points in the total score or a complete resolution of neurological deficits within 24 h after thrombolysis^9,14^.

### Statistical analysis

For continuous variables, summary statistics were expressed as the mean ± standard deviation ($\bar{X}$ ± SD) or median (interquartile range) (M [Q1, Q3]) depending on the distribution of statistical data. The categorical variables were expressed as n (%). Independent t-test was used for continuous variables with normal distribution, and Mann-Whitney U test was used for non-normal distribution. Comparisons between categorical variables were made using Pearson χ^2^ test or Fisher′s exact test. Spearman correlation analysis was performed to determine associations between variables. Univariable and multivariable binary logistic regression analyses were used to determine the independent risk factors of HT and ENI by obtaining odds ratios (OR) and 95% confidence intervals (CI). Receiver operating characteristic (ROC) curves were applied to evaluate the ability of ΔNLR in identifying HT and ENI. SPSS 23.0 software was used for statistical analysis. *P* < 0.05 were deemed statistically significant.

## Results

### Baseline characteristics

**Fig. 2:**
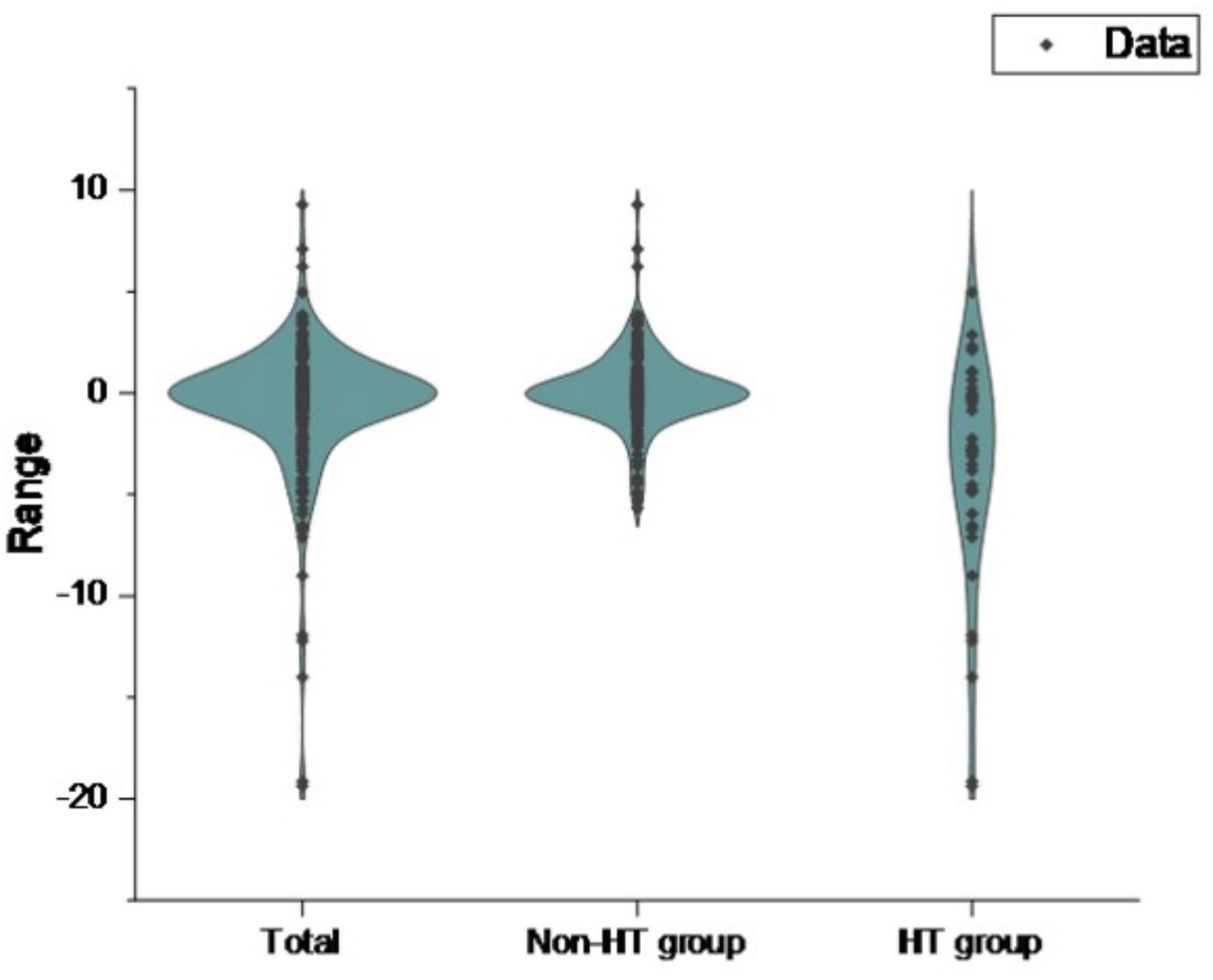
Violin plot of ΔNLR in HT group versus non- HT group and overall.

**Fig. 3:**
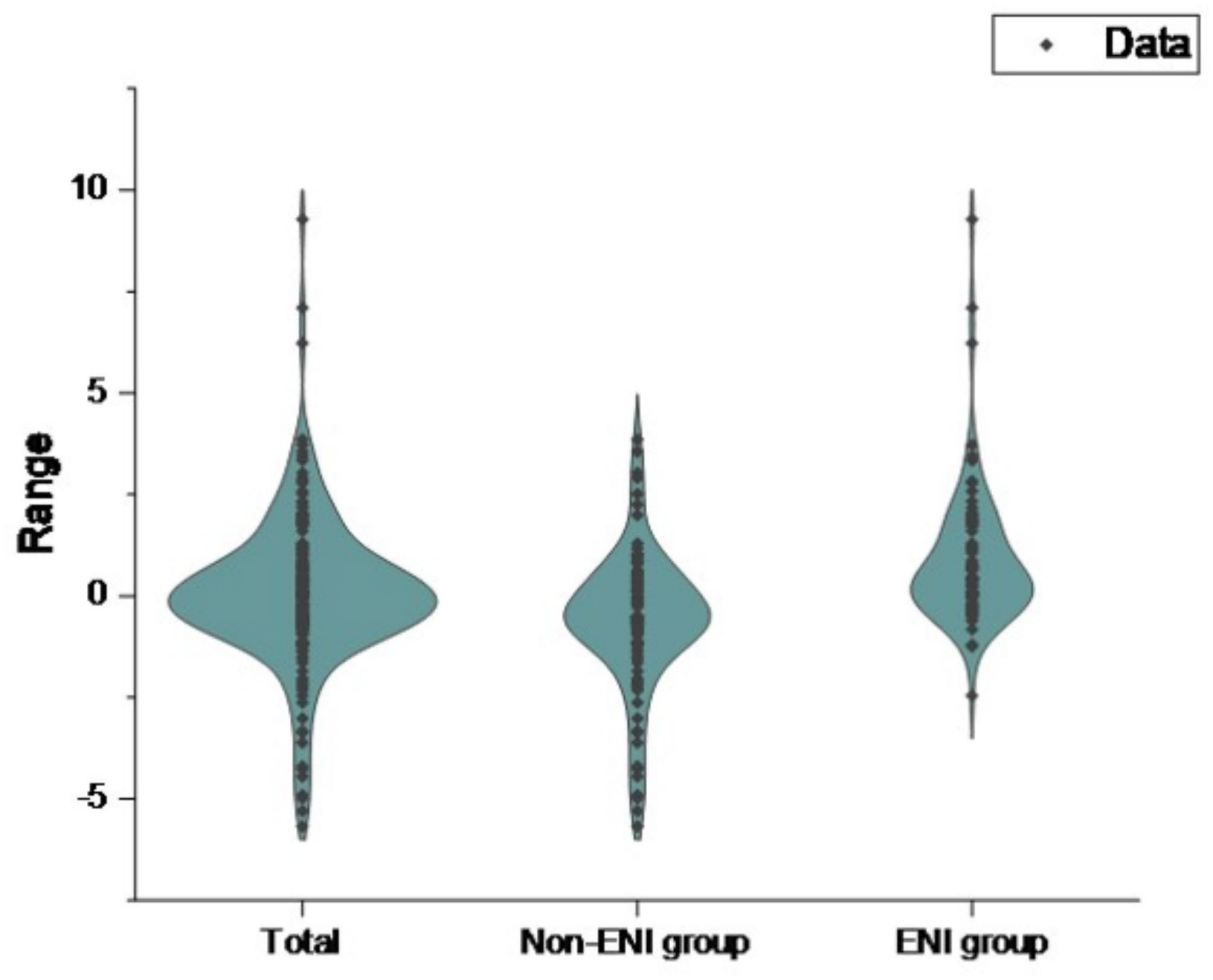
Violin plot of ΔNLR for ENI group versus non-ENI group and overall.

Among the 234 patients, 39 developed HT. According to the presence or absence of HT, the main baseline characteristics of patients were shown in Table 1; Fig. 2. Compared with the non-HT group, the HT group had higher levels of age, NIHSS score before IVT, baseline NLR and the proportion of patients with a history of atrial fibrillation (*P* < 0.05). In contrast, the levels of OTT and platelets were lower in those HT patients. The ΔNLR in HT group (-2.86[-5.95, -0.19]) was lower than that in non-HT group (0.02[-0.62, 0.79]) (*P* < 0.001). Other clinical parameters in the two groups were not statistically significant (*P* > 0.05). Of 195 patients who did not develop HT, 89 presented ENI. Based on the presence or absence of ENI, the main baseline characteristics of patients were shown in Table 2; Fig. 3. Thus, the ENI group had lower systolic blood pressure, larger baseline neutrophils, and larger baseline NLR compared with the non-ENI group (*P* < 0.05). The ΔNLR in ENI group (0.40[-0.05, 1.75]) was higher than that in non-ENI group (-0.58[-1.16, 0.29]) (*P* < 0.001). Other clinical parameters were not statistically significant between them (*P* > 0.05).

**Table 1:** Comparison of baseline data between the HT group and the non-HT group.

	Total(*n* = 234)	Non-HT group(*n* = 195)	HT group(*n* = 39)	*p*
Male, (%)	152(64.96)	130(66.67)	22(56.41)	0.220
Age, years	65[57,72]	64[56,72]	67[61,76]	0.015
ΔNLR	-0.07[-0.88,0.70]	0.02[-0.62,0.79]	-2.86[-5.95, -0.19]	<0.001
NIHSS score before IVT	5[3,8]	4[3,7]	11[4,16]	<0.001
OTT, minutes	163[110,210]	165[115,219]	130[95,178]	0.002
Systolic pressure, mmHg	150[136,165]	150[135,165]	151[136,167]	0.729
Diastolic pressure, mmHg	85 ± 16	85 ± 16	87 ± 13	0.507
BMI, kg/m^2^	25.15[22.60,27.34]	25.26[22.60,27.14]	24.82[22.66,27.68]	0.919
Baseline leukocyte,10^9/L	7.39[6.09,8.98]	7.34[6.08,8.86]	7.88[6.35,9.59]	0.467
Baseline neutrophils,10^9/L	4.46[3.46,5.93]	4.43[3.43,5.79]	5.48[3.80,6.59]	0.069
Baseline lymphocytes,10^9/L	1.98[1.57,2.64]	2.01[1.57,2.66]	1.86[1.44,2.55]	0.075
Platelets,10^9/L	210[178,251]	215[182,255]	190[154,230]	0.001
Baseline NLR	2.19[1.49,3.19]	2.14[1.45,3.00]	3.09[1.55,3.60]	0.014
AST, UL	24.78[21.06,30.52]	24.70[21.08,30.51]	25.04[20.00,30.88]	0.787
ALT, UL	24.30[18.00,31.20]	24.60[18.00,31.50]	22.09[16.10,31.06]	0.713
TBIL, umol/L	11.07[8.11,14.70]	11.00[8.23,14.64]	11.60[7.85,15.19]	0.625
Glu, mmol/L	7.45[6.04,9.54]	7.29[6.02,9.46]	8.08[6.48,11.20]	0.387
Urea, mmol/L	6.15[5.18,7.40]	6.08[5.19,7.31]	6.79[5.06,7.90]	0.191
Creatinine, umol/L	66.05[55.28,79.31]	66.30[54.80,79.30]	63.10[56.00,79.60]	0.984
CRP, mg/l	0.50[0.50,2.11]	0.50[0.50,2.08]	0.58[0.50,2.26]	0.402
Hypertension, (%)	151(64.53)	124(63.59)	27(69.23)	0.501
Diabetes, (%)	74(31.62)	63(32.31)	12(30.77)	0.900
Dyslipidemia, (%)	25(10.68)	23(11.79)	2(5.13)	0.344
CHD, (%)	43(18.38)	35(17.95)	8(20.51)	0.706
Atrial fibrillation, (%)	38(16.24)	22(11.28)	16(41.03)	<0.001
Smoking, (%)	82(35.04)	72(36.92)	10(25.64)	0.178
Alcoholism, (%)	65(27.78)	56(28.72)	9(23.08)	0.473

**Table 2:** Comparison of baseline data between the ENI group and the non-ENI group.

	Total(*n* = 195)	Non-ENI group(*n* = 106)	ENI group(*n* = 89)	*p*
Male, (%)	130(66.67)	72(67.92)	58(65.17)	0.684
Age, years	64[56,72]	63[56,70]	66[57,74]	0.250
ΔNLR	0.02[-0.62,0.79]	-0.58[-1.16,0.29]	0.40[-0.05,1.75]	<0.001
NIHSS score before IVT	4[3,7]	4[3,6]	5[3,8]	0.128
OTT, minutes	165[115,219]	163[109,210]	179[120,229]	0.096
Systolic pressure, mmHg	150[135,165]	156[138,172]	146[132,161]	0.020
Diastolic pressure, mmHg	85[74,97]	86[76,98]	81[72,94]	0.081
BMI, kg/m^2^	25.26[22.60,27.14]	25.95[22.57,27.85]	24.77[22.41,26.37]	0.081
Baseline leukocyte,10^9/L	7.34[6.08,8.86]	7.21[6.04,8.42]	7.55[6.12,9.26]	0.165
Baseline neutrophils,10^9/L	4.43[3.43,5.79]	4.11[3.12,5.26]	4.69[3.57,6.35]	0.026
Baseline lymphocytes,10^9/L	2.01[1.57,2.66]	2.22[1.67,2.68]	1.88[1.44,2.63]	0.057
Platelets,10^9/L	215[182,255]	211[178,257]	227[187,252]	0.283
Baseline NLR	2.14[1.45,3.00]	2.05[1.36,2.72]	2.41[1.61,3.45]	0.011
AST, UL	24.70[21.08,30.51]	23.99[20.40,30.86]	24.86[21.70,29.72]	0.555
ALT, UL	24.60[18.00,31.50]	23.55[17.93,33,13]	24.70[18.35,29.55]	0.916
TBIL, umol/L	11.00[8.23,14.64]	10.64[7.98,14.78]	11.16[8.58,14.59]	0.421
Glu, mmol/L	7.29[6.02,9.46]	7.55[6.08,9.68]	7.13[5.98,9.42]	0.459
Urea, mmol/L	6.08[5.19,7.31]	6.16[5.39,7.40]	5.93[4.97,7,29]	0.272
Creatinine, umol/L	66.30[54.80,79.30]	66.20[54.50,75.33]	66.60[55.10,80.35]	0.517
CRP, mg/l	0.50[0.50,2.08]	0.50[0.50,2.11]	0.67[0.50,2.10]	0.462
Hypertension, (%)	124(63.59)	68(64.15)	56(62.92)	0.859
Diabetes, (%)	63(32.31)	38(35.85)	25(28.09)	0.248
Dyslipidemia, (%)	23(11.79)	12(11.32)	11(12.36)	0.823
CHD, (%)	35(17.95)	18(16.98)	17(19.10)	0.701
Atrial fibrillation, (%)	22(11.28)	12(11.32)	10(11.24)	0.985
Smoking, (%)	72(36.92)	40(37.74)	32(35.96)	0.797
Alcoholism, (%)	56(28.72)	33(31.13)	23(25.84)	0.416

### Study on the correlation between NLR variation and HT

**Fig. 4:**
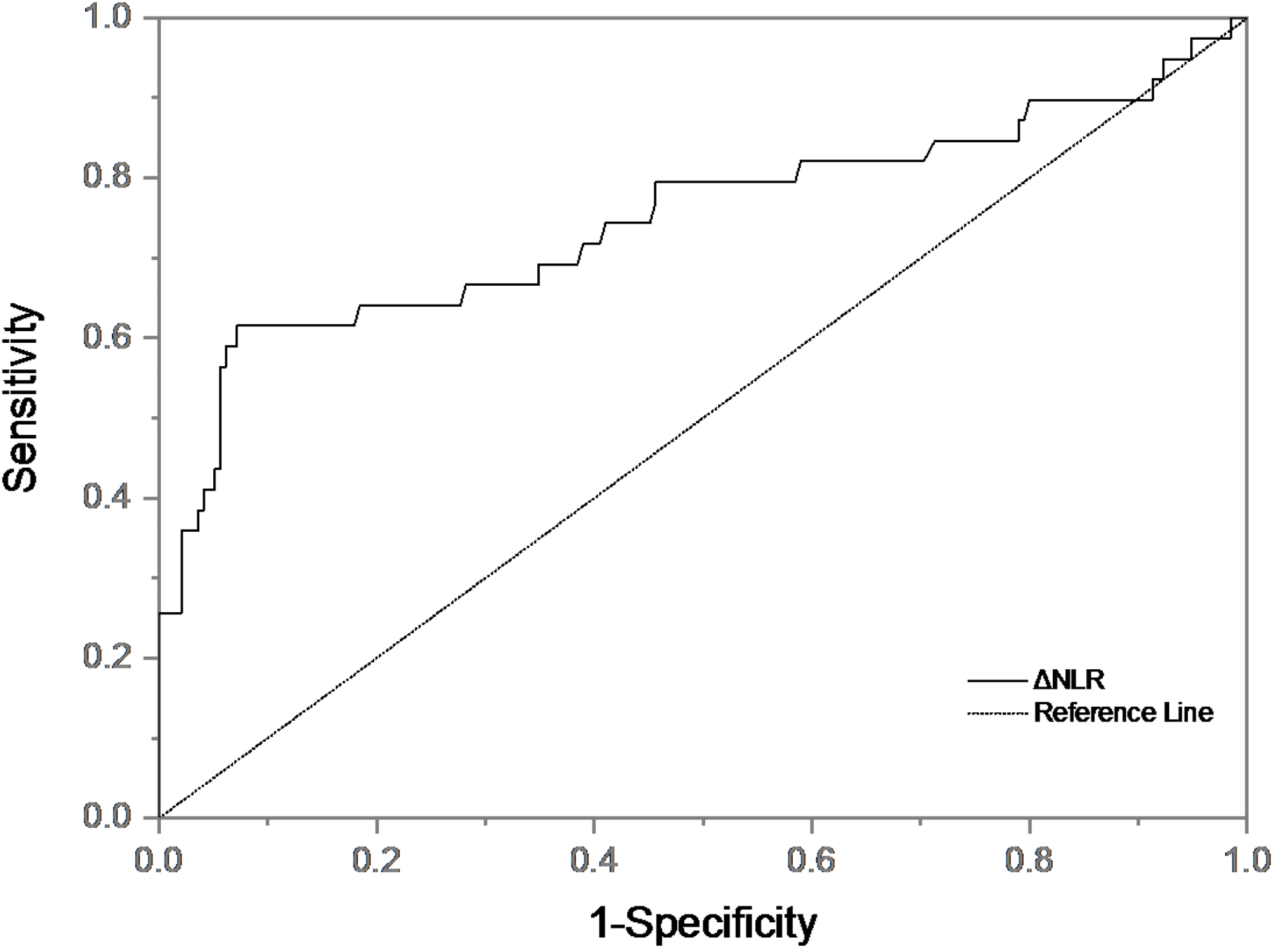
ROC curve of ΔNLR predicting HT.

There were statistically significant differences in age, ΔNLR, pre-thrombolytic NIHSS score, OTT, platelet, baseline NLR, and history of atrial fibrillation between the HT group and the non-HT group. Binary Logistic regression analysis was performed for the above indicators. We found that ΔNLR was an independent predictor of HT after adjusting for age, pre-thrombolytic NIHSS score, OTT, platelets, baseline NLR, and history of atrial fibrillation (Table 3). Patients with higher ΔNLR had a 0.580-fold(95% CI 0.467–0.720, *P* <0.001, Table 3) higher risk of developing HT. ΔNLR was included in ROC curve analysis (Fig. 4). The area under the curve (AUC) of ΔNLR differentiating HT was 0.749 [95%CI (0.644–0.854), *p* < 0.001)], with a sensitivity of 61.5% and specificity of 92.8%, indicating that ΔNLR had good predictive value (AUC > 0.7), and the cut-off value was − 2.26.

**Table 3:** Binary logistic regression analysis of HT group and non-HT group.

	OR	95% CI	*p*
Age	1.050	0.996–1.107	0.069
ΔNLR	0.580	0.467–0.720	<0.001
NIHSS score before IVT	1.033	0.925–1.154	0.565
OTT	0.988	0.978–0.997	0.012
Platelets,10^9/L	0.987	0.977–0.998	0.018
Baseline NLR	2.097	1.483–2.965	<0.001
Atrial fibrillation	1.895	0.517–6.940	0.335

### Correlation study between NLR variation and ENI

**Fig. 5:**
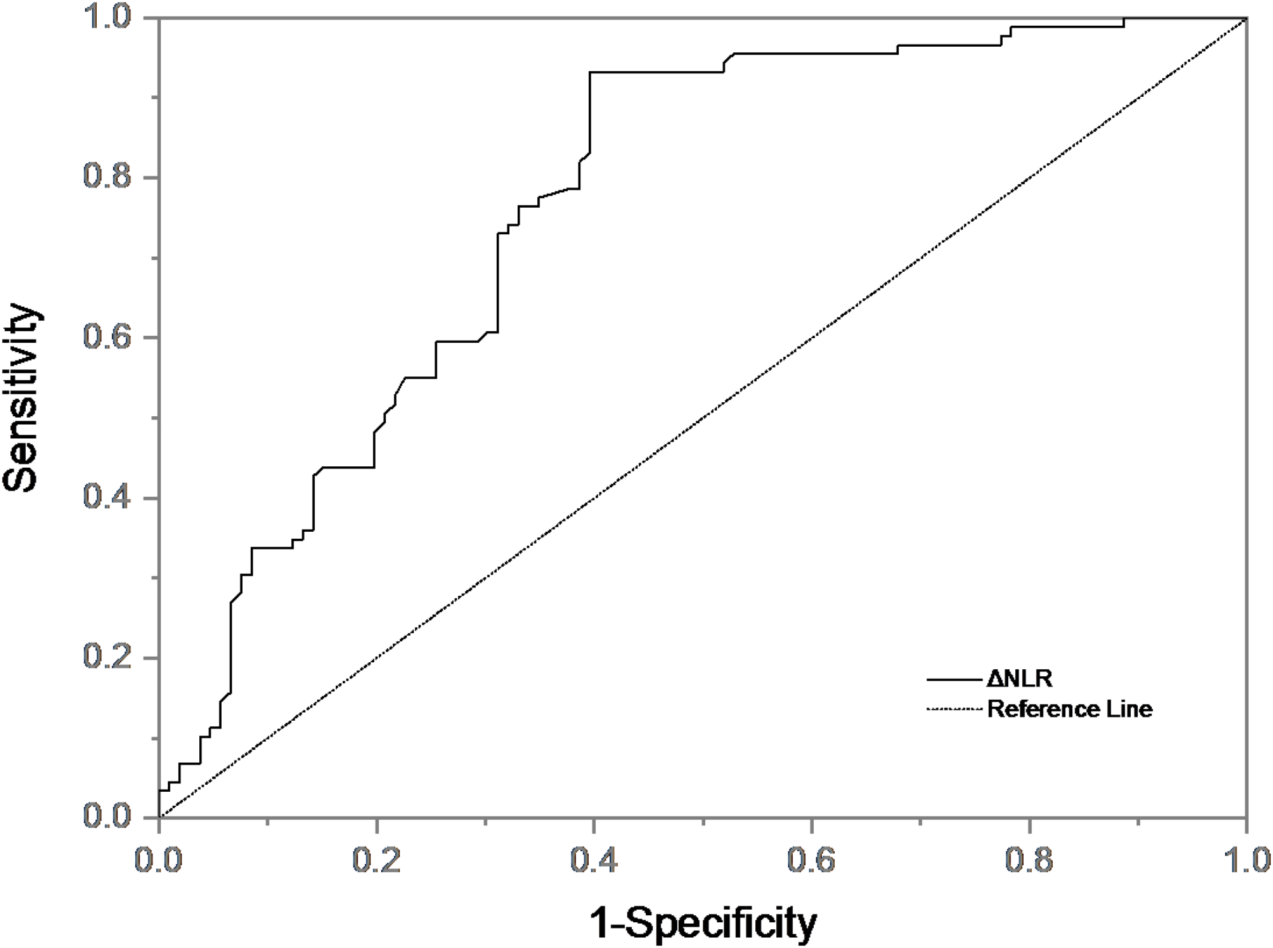
ROC curve of ΔNLR predicting ENI.

There were statistically significant differences in ΔNLR, systolic blood pressure, baseline neutrophils and baseline NLR between ENI group and non-ENI group. Binary Logistic regression analysis was performed for the above indicators. We found that ΔNLR was an independent predictor of ENI (Table 4). Patients with higher ΔNLR had a 2.369-fold(95% CI 1.629–3.444, *P* < 0.001, Table 4) higher risk of developing ENI. ΔNLR was included in ROC curve analysis (Fig. 5). ΔNLR distinguished ENI with an AUC of 0.768 [95%CI (0.72–0.835), *P* < 0.001)], suggesting that ΔNLR had good predictive value (AUC > 0.7). The cut-off value was − 0.49 with a sensitivity of 93.3% and specificity of 60.4%.

In order to further explore the relationship between ΔNLR and ΔNIHSS score, we conducted Spearman correlation analysis to assess the relationship between ΔNLR and ΔNIHSS score in AIS patients. It could be seen that ΔNLR was positively correlated with ΔNIHSS score of AIS patients (*r* = 0.289, *p* <0.001). And Spearman correlation analysis was conducted to assess the relationship between ΔNLR and 7-day mRS score in AIS patients. It could be seen that ΔNLR was negatively correlated with mRs score of AIS patients (*r*=-0.345, *p* <0.001).

**Table 4:** Binary logistic regression analysis of ENI group and non-ENI group.

	OR	95% CI	*p*
ΔNLR	2.369	1.629–3.444	<0.001
Systolic pressure	0.983	0.969–0.997	0.016
Baseline neutrophils	1.018	0.781–1.327	0.896
Baseline NLR	0.774	0.527–1.138	0.193

### Association of ΔNLR with early outcomes after IVT in patients with different TOAST classification

#### Association of ΔNLR with early outcomes after IVT in atherosclerotic and cardioembolic patients

**Fig. 6:**
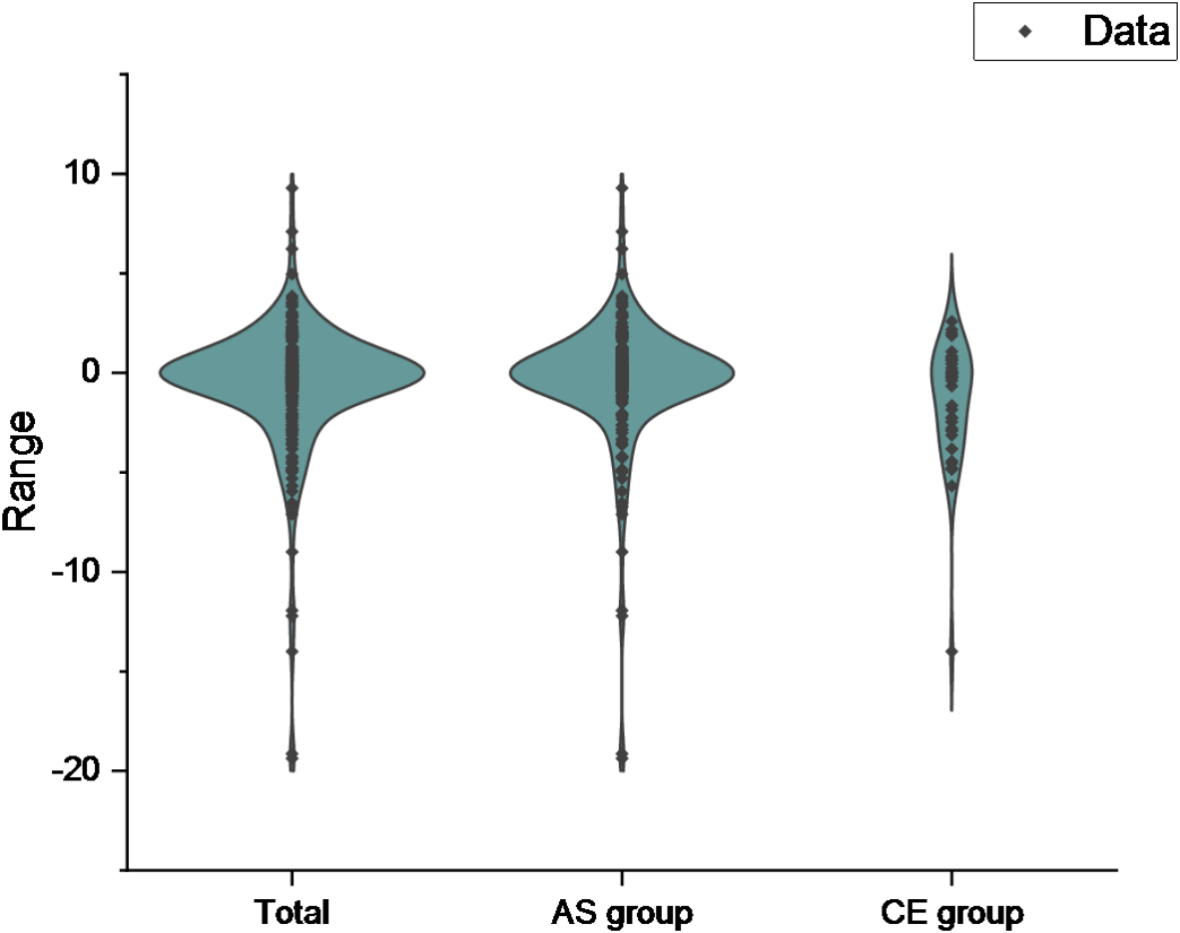
Violin plot of ΔNLR for AS group versus CE group and overall.

Among the 234 patients, 198 were atherosclerosis (AS) subtype and 36 were cardiac embolism (CE) subtype. The main baseline characteristics of the patients were shown in Table 5; Fig. 6. The incidence of HT in CE group (41.67%) was higher than that in AS group (12.12%) (*P* < 0.05). There was no significant difference in the incidence of ENI between the two groups (*P* > 0.05). Compared with AS group, CE group had older age, higher pre-thrombolytic NIHSS score, lower systolic blood pressure, fewer platelets, higher urea, higher CRP, and a greater proportion of patients with a history of atrial fibrillation (*P* < 0.05). The ΔNLR in CE group (-0.31[-2.94, 0.50]) was lower than that in AS group (-0.04[-0.75,0.75]) (*P* < 0.05). Binary Logistic regression analysis was carried out for the above indicators. We found that ΔNLR was not an independent factor between AS group and CE group (OR 0.987, 95% CI 0.822–1.185, *P* = 0.886, Table 6).

**Table 5:** Comparison of baseline data between AS group and CE group.

	Total(*n* = 234)	AS group(*n* = 198)	CE group(*n* = 36)	*p*
Male, (%)	152(64.96)	133(67.17)	19(52.78)	0.096
Age, years	65[57,72]	63[56,70]	73[64,80]	<0.001
ΔNLR	-0.07[-0.88,0.70]	-0.04[-0.75,0.75]	-0.31[-2.94,0.50]	0.039
NIHSS score before IVT	5[3,8]	4[3,7]	11[5,14]	<0.001
OTT, minutes	163[110,210]	163[110,215]	160[110.202]	0.653
Systolic pressure, mmHg	150[136,165]	151[138,166]	142[124,163]	0.024
Diastolic pressure, mmHg	85 ± 16	86 ± 15	82 ± 20	0.057
BMI, kg/m^2^	25.15[22.60,27.34]	25.31[22.91,27.50]	23.60[21.02,26.82]	0.070
Baseline leukocyte,10^9/L	7.39[6.09,8.98]	7.43[6.06,9.04]	7.22[6.43,8.80]	0.706
Baseline neutrophils,10^9/L	4.46[3.46,5.93]	4.51[3.43,5.90]	4.34[3.75,5.93]	0.417
Baseline lymphocytes,10^9/L	1.98[1.57,2.64]	2.00[1.57,2.65]	1.89[1.57,2.45]	0.328
Platelets,10^9/L	210[178,251]	214[181,251]	188[176,237]	0.049
Baseline NLR	2.19[1.49,3.19]	2.19[1.41,3.16]	2.24[1.78,3.44]	0.273
AST, UL	24.78[21.06,30.52]	25.00[21.02,30.94]	23.11[21.00,27.27]	0.310
ALT, UL	24.30[18.00,31.20]	24.62[18.05,31.05]	22.55[17.15,32.85]	0.755
TBIL, umol/L	11.07[8.11,14.70]	10.76[8.14,14.40]	12.37[8.07,18.50]	0.158
Glu, mmol/L	7.45[6.04,9.54]	7.46[6.02,9.59]	7.45[5.25,9.34]	0.966
Urea, mmol/L	6.15[5.18,7.40]	6.05[5.07,7.09]	7.41[5.79,9.06]	0.006
Creatinine, umol/L	66.05[55.28,79.31]	65.90[54.53,75.69]	70.60[56.05,81.44]	0.197
CRP, mg/l	0.50[0.50,2.11]	0.50[0.50,1.97]	1.17[0.50,2.64]	0.038
Hypertension, (%)	151(64.53)	128(64.65)	23(63.89)	0.930
Diabetes, (%)	74(31.62)	62(31.31)	12(33.33)	0.811
Dyslipidemia, (%)	25(10.68)	19(9.60)	6(16.67)	0.206
CHD, (%)	43(18.38)	34(17.17)	9(25.00)	0.265
Atrial fibrillation, (%)	38(16.24)	11(5.56)	27(75.00)	<0.001
Smoking, (%)	82(35.04)	74(37.37)	8(22.22)	0.080
Alcoholism, (%)	65(27.78)	59(29.80)	6(16.67)	0.106
HT, (%)	39(16.67)	24(12.12)	15(41.67)	<0.001
ENI, (%)	84(35.90)	75(37.88)	9(25.00)	0.138

**Table 6:** Binary logistic regression analysis of AS group and CE group.

	OR	95% CI	*p*
Age	1.031	0.978–1.086	0.255
ΔNLR	0.987	0.822–1.185	0.886
NIHSS score before IVT	1.072	0.966–1.189	0.191
Systolic pressure	0.982	0.959–1.005	0.129
Platelets	0.998	0.989–1.006	0.586
Urea	1.221	0.917–1.626	0.172
CRP	1.005	0.928–1.088	0.907
Atrial fibrillation	31.200	10.189–95.538	<0.001

#### Association of ΔNLR with early outcomes after IVT in patients with minor stroke of large artery atherosclerosis (LAA) subtype and small artery occlusion

**Fig. 7:**
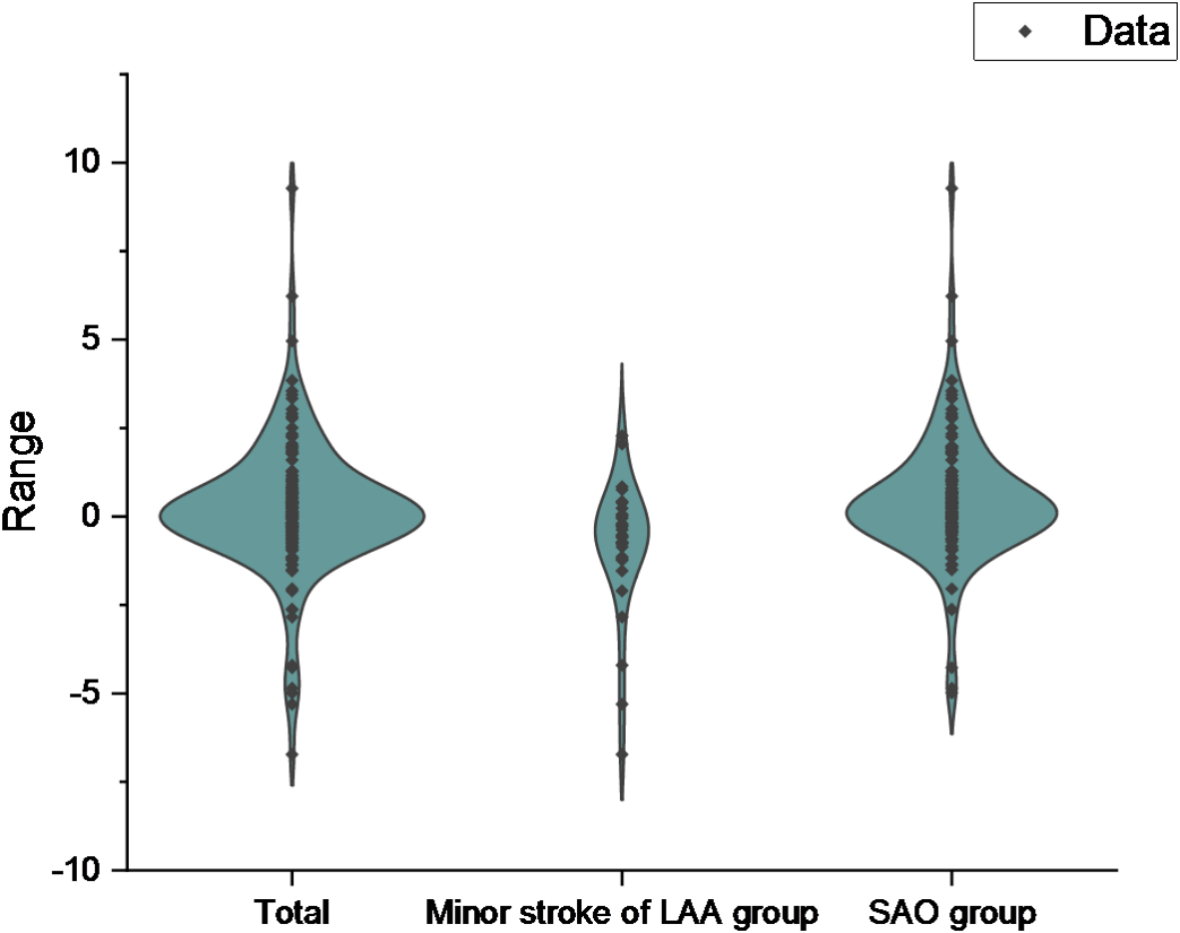
Violin plot of ΔNLR for minor stroke of LAA group versus SAO group and overall.

**Fig. 8:**
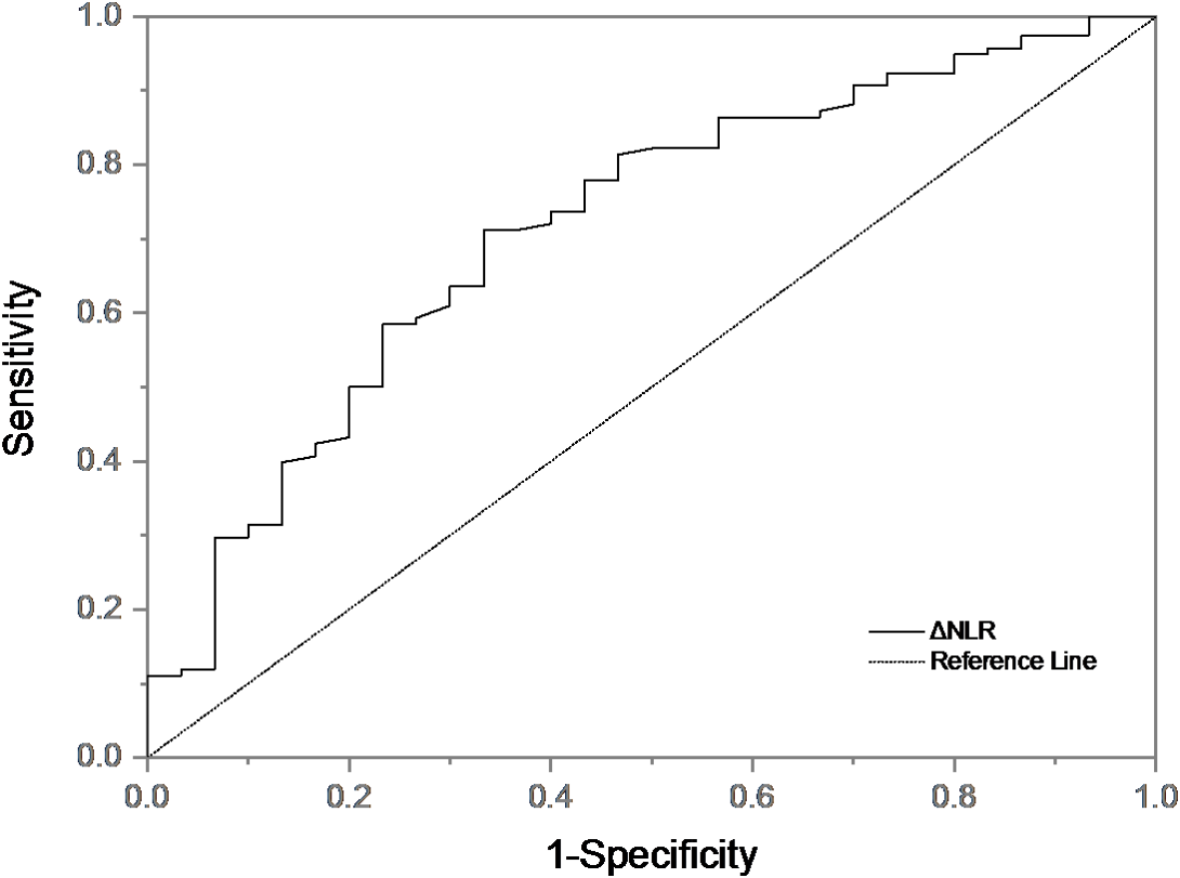
ROC curve of ΔNLR distinguishing minor stroke of LAA group and SAO group.

Among the 234 patients, there were 30 patients with minor stroke of LAA subtype (NIHSS score ≤ 3)^15^ and 118 patients with small artery occlusion (SAO) subtype. The main baseline characteristics of the patients were shown in Table 7; Fig. 7. The incidence of HT in the minor stroke of LAA group (20.00%) was higher than that in the SAO group (5.08%), and the incidence of ENI in the SAO group (39.83%) was higher than that in the minor stroke of LAA group (20.00%) (*P* < 0.05). There was no significant difference in NIHSS score before IVT between the two groups (*P* > 0.05). Compared with the minor stroke of LAA group, the SAO group had a younger age and a larger ALT (*P* < 0.05). The ΔNLR in SAO group (0.25[-0.30, 1.09]) was higher than that in minor stroke of LAA group (-0.55[-1.20,0.11]) (< 0.001). Binary Logistic regression analysis was performed on the relevant indicators. We found that, after adjusting for other factors, ΔNLR was an independent factor between the minor stroke of LAA group and the SAO group (OR 1.542, 95% CI 1.158–2.052, *P* = 0.003, Table 8). ΔNLR was included in ROC curve analysis (Fig. 8). The AUC of ΔNLR differentiating the minor stroke of LAA group from the SAO group was 0.716 [95%CI (0.613–0.819), *P* < 0.001)], the cut-off value was − 0.20, the sensitivity was 71.2%, and the specificity was 66.7%.

**Table 7:** Comparison of baseline data between minor stroke of LAA group and SAO group.

	Total(*n* = 148)	Minor stroke of LAA group(*n* = 30)	SAO group(*n* = 118)	*p*
Male, (%)	95(64.19)	18(60.00)	77(65.25)	0.592
Age, years	62 ± 11	66 ± 9	61 ± 11	0.028
ΔNLR	0.09[-0.52,0.89]	-0.55[-1.20,0.11]	0.25[-0.30,1.09]	<0.001
NIHSS score before IVT	3[2,5]	3[2,3]	3[1,5]	0.409
OTT, minutes	170[120,220]	180[130,233]	166[120,219]	0.305
Systolic pressure, mmHg	152[140,168]	159[143,175]	150[138,167]	0.123
Diastolic pressure, mmHg	87 ± 15	85 ± 12	87 ± 16	0.391
BMI, kg/m^2^	25.65[23.33,28.28]	25.21[23.47.27.83]	25.71[23.28,28.35]	0.862
Baseline leukocyte,10^9/L	7.32[6.08,8.61]	7.17[6.09,9.28]	7.42[6.00,8.59]	0.708
Baseline neutrophils,10^9/L	4.43[3.43,5.75]	4.12[3.46,5.26]	4.53[3.43,5.79]	0.497
Baseline lymphocytes,10^9/L	1.97[1.56,2.66]	2.21[1.67,2.72]	1.93[1.52,2.66]	0.269
Platelets,10^9/L	212[180,250]	217[163,256]	212[183,250]	0.554
Baseline NLR	2.21[1.41,3.16]	2.16[1.22,2.87]	2.22[1.53,3.22]	0.248
AST, UL	24.95[20.71,30.46]	23.98[18.41,31.90]	24.99[21.40,30.22]	0.545
ALT, UL	24.30[18.24,30.20]	20.27[16.08,27.04]	25.14[19.60,32.24]	0.011
TBIL, umol/L	11.07[8.11,14.58]	11.56[7.40,14.31]	10.91[8.31,14.82]	0.642
Glu, mmol/L	7.52[6.19,9.64]	7.67[6.14,9.74]	7.49[6.19,9.63]	0.968
Urea, mmol/L	6.05[5.10,7.09]	5.99[4.76,7.07]	6.05[5.20,7.10]	0.642
Creatinine, umol/L	64.15[53.43,75.07]	64.20[50.70,74.75]	64.15[54.15,75.98]	0.680
CRP, mg/l	0.50[0.50,1.61]	0.50[0.50,1.07]	0.50[0.50,1.71]	0.111
Hypertension, (%)	96(64.86)	22(73.33)	74(62.71)	0.277
Diabetes, (%)	50(33.78)	11(36.67)	39(33.05)	0.708
Dyslipidemia, (%)	16(10.81)	2(6.67)	14(11.86)	0.625
CHD, (%)	25(16.89)	7(23.33)	18(15.25)	0.292
Atrial fibrillation, (%)	4(2.70)	2(6.67)	2(1.69)	0.385
Smoking, (%)	54(36.49)	12(40.00)	42(35.59)	0.654
Alcoholism, (%)	48(32.43)	14(46.67)	34(28.81)	0.062
HT, (%)	12(8.11)	6(20.00)	6(5.08)	0.008
ENI, (%)	53(35.81)	6(20.00)	47(39.83)	0.043

**Table 8:** Binary logistic regression analysis of minor stroke of LAA group and SAO group.

	OR	95% CI	*p*
Age	0.953	0.909–0.999	0.044
ΔNLR	1.542	1.158–2.052	0.003
ALT	1.027	0.989–1.066	0.171

## Discussion

This study was the first time to confirm the correlation of ΔNLR with HT and ENI 24 h after IVT in AIS patients. The ΔNLR was calculated as follows: baseline NLR minus NLR after IVT. Therefore, if the variation of NLR was a positive value, it means that the NLR after thrombolysis was lower than the baseline; if the variation was a negative value, it means that the NLR after thrombolysis was higher than the baseline. In this study, it was found that NLR increased more significantly after IVT in patients with HT, and NLR decreased more significantly after IVT in patients with ENI. After adjusting for other factors, ΔNLR was an independent influence factor on HT and ENI after IVT. Moreover, ΔNLR was positively correlated with ΔNIHSS score of AIS patients (*r* = 0.289, *p* <0.001), and it was negatively correlated with Modified Rankin Scale (mRS) score at 7 days following IVT (*r*=-0.345, *p* <0.001). And the NLR decreased more significantly after IVT in patients with SAO subtype than that in minor stroke of LAA subtype. However, there was no significant difference in ΔNLR between patients of AS subtype and CE subtype.

The influence of neutrophils on HT and prognosis in AIS patients may be attributed to their roles in ischemic injury and disruption of the blood–brain barrier (BBB). As one of the earliest responding cells following AIS^5^, neutrophils can act on tight-junction proteins via matrix metalloproteinase-9 (MMP-9) to open the BBB, or they can be internalized into endothelial cells and affect the basement membrane^3^. Both mechanisms contribute to the increased incidence of HT following thrombolysis in AIS patients^16^. Previous study have proposed that in LAA subtype stroke, plasma neutrophil elastase (NE) may also be an important mediator of high neutrophil involvement in HT^17^. Additionally, other factors released by neutrophils after AIS, including reactive oxygen species (ROS) (e.g., superoxide, hypochlorous acid), interleukin-6 (IL-6), and various chemokines (e.g., CCL2, CCL3, CCL5), can not only affect the BBB and brain parenchyma^18^, but also impair the fibrinolytic system by stimulating the production of plasminogen activator inhibitor type 1 (PAI-1)^19^, thereby influencing patient prognosis.

Lymphocytes contribute positively to the prognosis of AIS patients following IVT. The lymphocyte count serves as a general health indicator, reflecting the effects of acute physiological stress^20^. It can indicate the stress response mediated by cortisol and the level of sympathetic activation^21^. An increase in lymphocyte count can reduce the production of pro-inflammatory cytokines, thereby mitigating ischemic injury and enhancing the incidence of ENI^22^. Studies have demonstrated that regulatory T cells (Tregs) and regulatory B cells (Bregs) exhibit protective effects in certain inflammatory diseases of the central nervous system^23–25^. This suggests that lymphocytes may protect against ischemic brain injury by suppressing inflammation, thereby improving the prognosis of AIS patients after IVT^6^. However, the specific mechanisms and the roles of various lymphocyte subtypes in HT and ENI following IVT in AIS patients remain to be further elucidated. This study focused on the variation of NLR, which comprehensively reflected the inflammatory role of neutrophils and the immune role of lymphocytes^26^. Neutrophil-mediated release of inflammatory cytokines may induce lymphocyte apoptosis^7^. Due to overactivation of neutrophils, the gap between neutrophils and lymphocytes could increase, resulting in significant changes in NLR.

TOAST classification is widely used internationally for AIS. Patients with CE stroke exhibit a higher incidence of HT compared to other subtypes, resulting in more severe brain injury and higher disability and mortality rates. This is associated with a high-inflammatory environment caused by complex mechanisms at the genetic, molecular, and cellular levels^27^. In this study, we observed that the CE group had a higher incidence of HT than the AS group, although ΔNLR was not identified as an independent influencing factor between these two groups. Compared with SAO stroke, patients with LAA cerebral infarction have higher NIHSS score at admission, more severe neurological impairment and worse clinical prognosis^28^. They were also more likely to develop HT. Feng, X^29^. et al. found that higher levels of neutrophil counts and NLR were associated with higher stress hyperglycemia ratio levels in AIS patients with LAA. They speculated that stress hyperglycemia may contribute to the progression of atherosclerosis by affected peripheral blood lymphocytes and neutrophils and disrupting BBB in patients with LAA^29^. In this study, we compared patients with minor LAA subtype stroke to those with SAO stroke. After balancing the pre-thrombolysis NIHSS scores across groups, we still observed that patients with minor LAA stroke had a higher incidence of HT, a lower incidence of ENI, and a lower ΔNLR. Patients in the minor LAA stroke group exhibited significantly higher NLR and more severe inflammatory responses after intravenous thrombolysis (IVT) compared to baseline. This difference between subtypes was independent of the baseline NIHSS score.

The main advantage of the study was that the blood samples of all patients were collected twice, which could reflect the changes of NLR, so as to observe the relationship of the dynamic changes of leukocyte with cerebral hemorrhage complications of thrombolytic therapy and the improvement of NIHSS score. We further examined the differences in ΔNLR before and after intravenous thrombolysis (IVT) among AIS patients with different subtypes using the TOAST classification. Additionally, this study employed strict exclusion criteria. Previous research has shown that infection is associated with increased white blood cell counts and early adverse neurological outcomes in AIS patients^30^ To minimize potential confounders, we excluded patients with infections. Nonetheless, our study has several limitations. First, it was a single-center study with a relatively small sample size. Therefore, multicenter studies with larger cohorts are needed to validate our findings. Second, we did not investigate the relationship between long-term prognosis and ΔNLR in patients with different TOAST classifications after IVT. Thus, the association between ΔNLR and long-term outcomes following IVT in AIS patients with different TOAST classifications needs further investigation.

## Conclusion

Our study demonstrated that both HT and ENI were associated with ΔNLR, which was an independent influencing factor for HT and ENI following IVT. Specifically, the ΔNLR in the SAO group was higher than that in the minor LAA group. Therefore, ΔNLR, in conjunction with baseline NLR, can be used to assess the risk of HT. Moreover, ΔNLR may serve as a feasible strategy to monitor and predict the outcomes of thrombolytic therapy in AIS patients.

## Data Availability

All data generated or analyzed during this study are included in this published article [and its supplementary information files].
